# Address Delivered before the Maryland State Dental Association, February 28th, 1889

**Published:** 1890-02

**Authors:** Wm. A. Mills


ARTICLE VII.

### ADDRESS DELIVERED BEFORE THE MARYLAND STATE DENTAL ASSOCIATION, FEBRUARY 28TH, 1889.

BY WM. A. MILLS, D. D. S.

GENTLEMEN:—As president, we welcome you in the name of Baltimore City, and the Maryland State Dental Association, on this, our Sixth Annual Meeting.

Many of you know the association is a legally authorized body, having been duly incorporated, under the laws of the State of Maryland, from its first meeting in the year 1884. Its objects as set forth in the Constitution, viz.: "The objects of this association shall be to promote professional and friendly intercourse, to develope dental literature, and to encourage a high standard of dental education." The great importance of this meeting cannot be over-estimated. It consists of dentists from all sections of the State, representing a profession of about three hundred members. By our co-operative efforts, sincerely and earnestly applied, we can give strength and influence to the dental profession in the State of Maryland.

In the past, little more has been done, than to declare the *purpose* of the association, prescribe the *requisites for membership*, *elect officers* and appoint *committees for special fields of labor*. At the last meeting, however, many were present prominent in the profession, who were apparently convinced of the prospective utility of the Association. They separated with an evident and enthusiastic purpose to assist in accomplishing the mission of the Association. It now remains for us to act with harmony, diligence and wisdom, so that our organization may become a powerful agency for good to our chosen profession, and entitle us to the gratitude of fellow men ; as, in other branches of science, the true student seeks the well-being and happiness of mankind. Not from any professional gain does he seek to hide the discoveries he makes; but, on the contrary, by associations such as this, he gladly imparts these discoveries, freely as possible to others who may use them for the happiness of men ; and in these meetings, we gain not only by the interchange of thought, but by personal influence and example of our noblest associates. How great and lasting is that influence, we rarely realize. " The man of Knightly fidelity and lofty patience never fails to leave his impress."

Perhaps it is not necessary, or even expected of us, that we should enumerate the many topics of discussions which will naturally suggest themselves to the minds of each member present; or have we the time to do so, as they could not receive at this meeting adequate consideration. It may be well, however, for us to call your attention to the more important.

DENTAL ETHICS.

You will pardon us for calling your attention to the relation the members bear to this association.

Members should be furnished with copies of the Constitution, By-Laws and Code of Ethics, adopted by the association, and strict compliance with the laws enforced. Independence of action by a member, in violation of the law, or disregard thereof, creates unrest, dissatisfaction, and failure. Again : There can be no doubt in the minds of the members present that fraternal fellowship is essential. Earnest, zealous and generous rivalry is to be expected, and encouraged, but it should be conducted with a scrupulous regard for the rights and integrity of others. While a practitioner has the right, when he believes a patient cannot afford to pay the regular fees, to diminish a bill, yet it should be considered *un-professional* to reduce the standard fees with a view to mercenary competition.

STANDARD OF THE PROFESSION.

We call your especial attention to the standard of the Dental Profession in the State of Maryland. Is not the primary cause, "The lack of information among the people pertaining to the profession as a *specialty* of medicine?" We do not say "among the masses," as every practitioner knows that this ignorance is not confined to these, but to all classes, a few, so called, intelligent physicians not excepted.

Are not we responsible for this condition of things? Have we, as faithful practitioners, done all in our power to enlighten our patients, and those with whom we come in contact? Many practitioners have stood beside their patients for years, as faithful operators and scientific men, but notwithstanding, nearly all have failed to make good their claim of *Teachers of the People.*

FEES.

We would recommend as one means of improving the standard, the adoption by this association, of a "schedule of fees," not to be adsolutely binding upon members, but as a guide for them, and as a paper of authority for their patients.

DENTAL HYGIENE.

We recommend as another, that some action be taken to have children in the public schools instructed in subjects pertaining to dentistry. As the cause of temperance is growing by careful reasoning, and scientific teaching in the public schools, so would the work we so ardently endeavor to accomplish be much simplified, aided and abetted, by the properly educated school-children. Lectures by competent practitioners should be delivered in the public schools, and where physiology has been introduced, the popular method of teaching by the sense of the eye, with illustrated drawings made by the pupils themselves.

AUXILIARY MEETINGS.

During the year, two auxiliary meetings have been held in this city. The attendance at each meeting being thirty or forty of the dentists of Baltimore city and county. That the meetings were appreciated by those in attendance, was guaranteed by the fact that many made the statement: they intended joining the parent organization, at its next annual meeting.

These meetings could be brought to a successful issue, but the preparation and money necessary, call for a separate organization.

BENEFICIARY SCHOLARSHIP.

In our official capacity we have received several applications for the Beneficiary Scholarship of the association. We have issued the same to Mr. ———— who brought us certificates of his academic, moral and dental qualifications. He also wrote out in full, and then signed, our Code of Ethics.

We thank you, gentlemen, for your kind attention.

